# Correlation of Cervical Cancer Mortality with Fertility, Access to Health Care and Socioeconomic Indicators

**DOI:** 10.1055/s-0039-1683859

**Published:** 2019-03-25

**Authors:** Diama Bhadra Vale, Catherine Sauvaget, Raul Murillo, Richard Muwonge, Luiz Carlos Zeferino, Rengaswamy Sankaranarayanan

**Affiliations:** 1 International Agency for Research on Cancer, Section of Early Detection and Prevention, Lyon, France; 2 Department of Tocoginecology, Universidade Estadual de Campinas, Campinas, SP, Brazil

**Keywords:** cervical cancer, cancer mortality, fertility, human development index, health disparities, câncer do colo do útero, mortalidade por câncer, fertilidade, índice de desenvolvimento humano, disparidades em saúde

## Abstract

**Objective** The present study aimed to examine which development indicators are correlated with cervical cancer (CC) mortality rates in Brazil.

**Methods** This was an ecological study that correlated mortality rates and indicators, such as human development index (HDI), gross domestic product (GDP) per capita, illiteracy rate, fertility rate, screening coverage, proportion of private health insurance use, density of physicians, and density of radiotherapy centers. The mortality rates were obtained from the Brazilian national registry, while the indicators were based on official reports from the Ministry of Health. Univariate and multivariate linear regression was used.

**Results** Among the states of Brazil, the average age-specific CC mortality rate from 2008 to 2012 varied from 4.6 to 22.9 per 100,000 women/year. In the univariate analysis, HDI, proportion of private health insurance use, density of physicians, and density of radiotherapy centers were inversely correlated with the mortality rates. Fertility rate was positively correlated with the mortality rates. In the multivariate analysis, only fertility rate was significantly associated with the CC mortality rate (coefficient of correlation: 9.38; 95% confidence interval [CI]: 5.16–13.59).

**Conclusion** A decrease in the fertility rate, as expected when the level of development of the regions increases, is related to a decrease in the mortality rate of CC. The results of the present study can help to better monitor the quality assessment of CC programs both among and within countries.

## Abstract (pt)

**Resumo:**

**Objetivo** O presente estudo teve como objetivo examinar quais indicadores de desenvolvimento estão correlacionados com as taxas de mortalidade por câncer do colo do útero no Brasil.

**Métodos** Este foi um estudo ecológico que correlacionou as taxas de mortalidade com indicadores como índice de desenvolvimento humano (IDH), produto interno bruto (PIB) per capita, taxa de analfabetismo, taxa de fertilidade, cobertura do rastreamento, proporção do uso do seguro privado de saúde, densidade de médicos e densidade de centros de radioterapia. A fonte das taxas de mortalidade foi o registro nacional, enquanto que os indicadores foram baseados em relatórios oficiais do Ministério da Saúde. Foi utilizada regressão linear univariada e multivariada.

**Resultados** Entre os estados, a taxa média de mortalidade específica por idade por câncer do colo do útero de 2008 a 2012 variou de 4.6 a 22.9 por 100.000 mulheres/ano. Na análise univariada, foram inversamente correlacionadas com as taxas de mortalidade: IDH, proporção do uso do seguro privado de saúde, densidade de médicos e densidade de centros de radioterapia. A taxa de fertilidade foi positivamente correlacionada com a mortalidade. Na análise multivariada, apenas a taxa de fertilidade foi significativamente associada à taxa de mortalidade por câncer do colo do útero (coeficiente de correlação: 9,38; índice de confiança [IC] 95%: 5,16–13,59).

**Conclusão** A diminuição da taxa de fertilidade, como esperado quando o nível de desenvolvimento das regiões aumenta, está relacionada a uma diminuição da taxa de mortalidade por câncer do colo do útero. Os resultados do presente estudo podem ajudar a monitorar melhor a avaliação da qualidade dos programas de câncer do colo do útero nos países tanto interna quanto externamente.

## Introduction

Cervical cancer (CC) is a common malignancy in women from developing countries. The main risk factor is a persistent infection with a high-risk human papilloma virus (HPV).^1^ Several cofactors may play a relevant role in the natural history of the disease, acting by facilitating the acquisition of HPV, favoring persistent infection or leading to progression from precursor to invasive lesions. Among the cofactors, parity has been consistently associated with an increased risk of CC.^2^ ^3^ ^4^ A high number of sexual partners and young age at the first sexual intercourse may influence the risk of acquisition of infection by HPV.^1^ Smoking, HIV infection, and/or immune deficiency also influence HPV acquisition and its persistence in the cervix, in addition to favoring the progression from precursor to invasive lesions.^5^

Organized CC screening has reduced the burden of the disease in many developed countries.^6^ Opportunistic screening has led to a certain control of the disease,^7^ coincident with improvements in socioeconomic conditions and with reductions in parity. Only few studies have addressed the association between development determinants and CC rates.^8^

In Brazil, CC is the third most common female neoplasia.^9^ There is a huge variation in rates among regions, reflecting their different levels of development.^9^ ^10^ ^11^ A guideline recommends screening women with cytology every 3 years from 25 to 64 years old.^12^ The screening is opportunistic, that is, it lacks an invitational strategy, and the quality assurance of the program is weak. Health care is free of charge to all Brazilian citizens, although high-income people usually co-use private health insurance. The quality of data varies among regions.

In the present study, in order to better monitor CC control actions we have corrected the CC mortality rates, and then we have correlated them with development indicators. We aimed to estimate which indicators were related to CC mortality at a population level.

## Methods

This was an ecological study. The CC mortality rates were estimated through the national mortality database from the Health Surveillance Department of the Ministry of Health.^13^ Population data were obtained from the national census and from projections from the Brazilian Institute of Geography and Statistics (IBGE, in the Portuguese acronym).^14^ The mortality rates were corrected in order to reduce the deficiencies in the quality of the reports. The indicators used reflected aspects of health care and socioeconomic development.

All of the recorded deaths from 2008 to 2012 were analyzed. Mortality data in Brazil are based on death certificates using the International Classifications of Disease (ICD-10) codes. Age-specific mortality rates by federal units were calculated using as numerator the number of deaths, and as denominator the population for the same age, federal unit, and year. The mortality rates were age-adjusted by the direct method using the world standard population (1960), and showed as average for the period studied (2008-2012). We have corrected the mortality rates based on the methodology suggested by Gamarra et al,^15^ reallocating proportionally the ill-defined causes of death (ICD-10 R-00 to R-99) among cancer and other causes of death (ICD-10 codes A to Q), except injury. The methodology for the correction is described by Vale et al.^11^

The development indicators were obtained from a period prior to 2008–2012 in order to consider the temporal relation between the indicators and the oucome (mortality).

Data on Human Development Index (HDI) were obtained from the 2000 report of the Brazilian office of the United Nations Development Program (UNDP-Brazil).^10^ This index is a composite factor that combines life expectancy, education, and per capita income. A high HDI is associated with a better human development level. The UNDP-Brazil estimates the HDI by municipalities, by federal units, and by regions.

Data on the availability of radiotherapy centers were used as a measure of access to cancer care and were obtained from the Brazilian National Commission of Nuclear Energy (CNEN, in the Portuguese acronym).^16^ Data were available only for the year of the analysis (2015). To obtain the density of radiotherapy centers per 1 million persons, we have used the IBGE population of the same year of the available data.

The remaining indicators were obtained from the Indicators and Basic Data for Brazil (IDB, in the Portuguese acronym),^17^ an annual publication based on census or national surveys of the Ministry of Health that presents relevant information for surveillance based on seven main domains: demographic, socioeconomic, mortality, morbidity, risk factors, resources, and health coverage.

In the IDB, the gross domestic product (GDP) per capita in 2000 was the ratio between the GDP of each federal unit and the population. The conversion rate for the Brazilian currency in July 2017 was of USD 1.00 = ∼ BRL 3.20. Illiteracy in 2000 was the proportion of the population > 15 years old that could not read or write simple notes. The fertility rate in 2000 was defined as the average of live births per women aged between 15 and 49 years old. Cervical cancer screening coverage in 2003 was the proportion of women aged between 25 and 59 years who underwent at least 1 screening test in the previous 3 years. Private health insurance in 2003 was the proportion of the population with access to it. The source on IDB for screening coverage and for private health insurance was the National Household Survey from the IBGE, based on self-reported data.^18^ The density of physicians was the number of active physicians per 1,000 persons in 2001, based on annual reports from the Brazilian Council of Medicine (CFM, in the Portuguese acronym).

Univariate and multivariate linear regression models were used to assess the effect of the different indicators on the mortality rates. The coefficients from the regression model and their 95% confidence intervals (CIs) were used to determine the direction of the effect for each factor on the CC mortality rates. A negative coefficient indicated a decreasing effect, whereas a positive coefficient meant an increasing effect on the mortality rates. The effects were concluded at a level of significance of 5%. In the main multivariate regression analysis, factors with a *p*-value < 0.05 were included. We have also performed a secondary analysis including only factors with a *p*-value ≤0.02. All of the analyses were performed using the software Stata, Version 13.0 (StataCorp LLC, College Station, TX, USA).

The Institutional Review Board of the School of Medicine of the Universidade Estadual de Campinas (Unicamp, in the Portuguese acronym), Campinas, state of São Paulo, Brazil, approved the present study as part of a research project evaluating the CC control program in Brazil, in collaboration with the International Agency for Research on Cancer (IARC). Informed consent was not needed since it was an analysis of aggregated data.

## Results

Table 1 shows the CC mortality rates and the demographic, socioeconomic, and selected health indicators in Brazil. The corrected age-specific cervical cancer mortality rate per 100,000 women/year (average rates from between 2008 and 2012) varied from 4.6 in the state of São Paulo (SP) to 22.9 in the state of Amazônia (AM).

**Table 1:** Cervical cancer mortality and demography, socio-economic and other health indicators in Brazil

Region	State	Cervical cancer mortality^a^	Human development index^b^	Gross domestic product per capita^c^	Illiteracy rate^d^	Fertility rate^e^	Screening coverage^f^	Private health insurance^g^	Density of Physicians^h^	Density of radiotherapy centers^i^
**North**	**AC**	9.42	0.517	3.86	23.60	2.97	70.20	18.10	0.60	0.00
	**AM**	22.88	0.515	5.96	14.96	3.40	68.16	13.50	0.60	10.16
	**AP**	15.02	0.577	4.94	11.57	3.61	72.85	16.20	0.42	0.00
	**PA**	13.41	0.518	3.08	15.92	3.15	71.73	16.70	0.51	4.89
	**RO**	8.29	0.537	4.31	12.16	2.73	85.06	9.60	0.59	11.31
	**RR**	12.69	0.598	5.48	12.33	3.66	71.25	18.60	0.10	0.00
	**TO**	10.86	0.525	3.17	17.82	2.92	69.97	7.10	0.72	6.60
**Northeast**	**AL**	9.22	0.471	2.75	31.73	3.16	47.68	8.40	0.95	8.98
	**BA**	6.60	0.512	3.56	21.99	2.50	69.09	13.30	0.86	5.26
	**CE**	7.77	0.541	3.04	24.95	2.81	69.27	11.80	0.75	4.49
	**MA**	13.46	0.476	2.11	27.09	3.22	60.33	6.80	0.45	2.90
	**PB**	6.64	0.506	2.71	28.19	2.53	60.94	12.50	0.94	5.03
	**PE**	8.03	0.544	3.40	22.97	2.49	72.91	15.70	1.09	5.35
	**PI**	9.86	0.484	2.13	29.16	2.65	75.40	10.80	0.60	6.24
	**RN**	6.66	0.552	3.28	23.85	2.54	72.92	10.80	0.90	2.91
	**SE**	10.44	0.518	3.66	23.72	2.75	64.85	13.60	0.85	8.92
**Midwest**	**DF**	6.45	0.725	22.66	5.35	2.19	75.42	32.20	2.73	13.72
	**GO**	7.49	0.615	5.25	11.21	2.24	77.68	23.40	1.10	6.05
	**MT**	8.18	0.601	5.94	11.32	2.46	67.73	17.20	0.69	6.12
	**MS**	8.83	0.613	5.45	10.60	2.10	81.97	29.70	1.03	11.32
**Southeast**	**ES**	7.31	0.640	7.51	10.82	2.05	78.79	24.70	1.39	7.63
	**MG**	5.13	0.624	5.62	11.32	2.22	70.54	25.40	1.40	11.98
	**RJ**	7.21	0.664	9.71	6.13	2.07	76.02	30.10	2.96	13.90
	**SP**	4.62	0.702	11.45	6.14	2.07	81.22	38.20	1.97	15.54
**South**	**PR**	6.50	0.650	7.23	8.93	2.09	73.91	24.10	1.24	13.44
	**RS**	5.91	0.664	8.03	6.21	2.11	75.65	31.90	1.82	16.89
	**SC**	5.93	0.674	8.09	5.83	2.04	80.38	27.20	1.13	14.66

Abbreviations: AC, Acre; AL, Alagoas; AP, Amapá; AM, Amazonas; BA, Bahia; CE, Ceará; DF, Distrito Federal; ES, Espírito Santo; GO, Goiás; MA, Maranhão; MT, Mato Grosso; MS, Mato Grosso do Sul; MG, Minas Gerais; PA, Pará; PB, Paraíba; PR, Paraná; PE, Pernambuco; PI, Piauí; RJ, Rio de Janeiro; RN, Rio Grande do Norte; RS, Rio Grande do Sul; RO, Rondônia; RR, Roraima; SC, Santa Catarina; SP, São Paulo; SE, Sergipe; TO, Tocantins.

a Corrected age-specific standardized cervical cancer mortality rate, average of 2008-2012.

b Human development index in 2000.

c Gross domestic product per capita in 2000, per BRL 1,000.00.

d Illiteracy rate in 2000, proportion of the population > 15 years old that cannot write and read.

e Fertility rate in 2000, average of live births per women between 15 and 49 years old.

f Screening coverage in 2003, proportion of women between 25 and 59 years old who underwent at least 1 screening test in the last 3 years.

g Private health insurance in 2003, proportion of the population with access to private health insurance.

h Density of physicians in 2001, rate of physicians per 1,000 persons.

i Density of radiotherapy centers in 2015, medical facilities with radiotherapy per 1,000,000 persons.

All of the federal units in the North and Northeast regions had HDIs < 0.6. The lowest GDP per capita observed was BRL 2,110 (∼ USD 600) in Maranhão (MA), and the highest was BRL 22,660 (∼ USD 6,500.00) in the Federal District (DF). The South and Southeast regions showed the highest GDP per capita. The northeast region had the higher proportion of illiteracy in the population. The fertility rate varied from 2.04 in Santa Catarina (SC) to 3.66 in Roraima (RR). These rates were lower in the South and Southeast regions, and higher in the North region.

The screening coverage of women aged between 25 and 59 years old was > 60% in all federal units. The proportion of private health insurance use was > 20% in the federal units from the South and Southeast regions, and < 20% in all federal units from the North and Northeast regions. The density of physicians was > 1 physician per 1,000 persons in the South and Southeast regions, and < 1 in the North and Northeast region, except for one federal unit (Pernambuco [PE]). Three federal units in the North region—Acre (AC), Amapá (AP), and Roraima (RR)—had no licensed radiotherapy centers in 2015. The highest density of radiotherapy centers was 16.89, in Rio Grande do Sul (RS) (Table 1).

Table 2 shows the correlation of indicators with the corrected CC mortality rates. In the univariate regression analysis, increasing fertility rates were associated with increasing mortality rates. Human development index, proportion of population using private health insurance, density of physicians, and density of radiotherapy centers were inversely associated with increasing CC mortality rates. After the adjustment, for all variables significantly associated with CC mortality at the univariate analysis, only fertility rate remained associated with CC mortality rates (coefficient of correlation: 7.54, 95% CI: 4.48-10.59; *p *< 0 .001) (Table 2). We also performed the multivariate analysis excluding density of radiotherapy centers (*p*-value = 0.04 in the univariate analysis), and fertility rate still remained significantly correlated (coefficient of correlation 6.75; 95% CI 3.74–9.76; *p* < 0.001).

**Table 2:** Regression analysis for correlation of cervical cancer mortality^a^ and indicators in Brazil

	Univariate analysis			Multivariate analysys		
Indicator	Coefficient	95% CI	*p-value*	Coefficient	95% CI	*p-value*
Human development index^b^	−0.03	−0.04; −0.01	0.01	−0.01	−0.05; + 0.02	0.50
Gross domestic product per capita^c^	−0.26	−0.62; +0.11	0.17			
Illiteracy rate^d^	+0.09	−0.10; +0.28	0.34			
Fertility rate^e^	+ 6.08	+ 4.16;+ 8.00	< 0.001	+ 7.54	+ 4.48; + 10.59	< 0.001
Screening coverage^f^	−0.14	−0.34;+ 0.06	0.17			
Private health insurance^g^	−0.20	−0.36;−0.04	0.02	+0.12	−0.17; + 0.41	0.85
Physician density^h^	−3.06	−5.09;−1.03	0.01	−0.59	−3.14; + 1.95	0.63
Density of radiotherapy centres^i^	−0.31	−0.60;−0.01	0.04	+ 0.25	−0.06; + 0.58	0.11

Abbreviations: CI, confidence interval.

a Corrected age-specific standardized cervical cancer mortality rate, average of 2008-2012;

b Human development index in 2000.

c Gross domestic product per capita in 2000, per BRL 1,000.00.

d Illiteracy rate in 2000, proportion of the population > 15 years old that cannot write and read.

e Fertility rate in 2000, average of live births per women between 15 and 49 years old.

f Screening coverage in 2003, proportion of women between 25 and 59 years old who underwent at least 1 screening test in the last 3 years.

g Private health insurance in 2003, proportion of the population with access to private health insurance.

h Density of physicians in 2001, rate of physicians per 1,000 persons.

i Density of radiotherapy centers in 2015, medical facilities with radiotherapy per 1,000,000 persons.

Coefficient of correlation: (+) means increasing effect and (-) means decreasing effect.

## Discussion

In the present study, fertility was the strongest indicator associated with CC mortality rates, even if the availability and efficiency of health care may also have played an important role. Indicators of access to health care were associated with mortality rates only in the univariate analysis. Access and fertility may be influenced by the level of development of the regions.^8^ ^15^ ^19^ The corrected mortality rates varied in Brazil.

The number of live births per women is consistently associated with CC, even when adjusted for sexual factors, history of Pap smear, and HPV positivity.^2^ ^20^ The probable mechanism by which fertility may affect the risk of cervical cancer is by facilitating the HPV infection and persistence in the transformation zone of the cervix.^1^ ^5^ ^20^ Hormonal changes in pregnancy lead to eversion of the squamous-columnar junction and metaplasia, triggering HPV infection and carcinogenesis. Cervix traumas during labor favor the maintenance of the eversion due to anatomical changes.

There are some quite reasonable evidences favoring these arguments. The risk of CC is strongly associated with the number of full term pregnancies, and not so to parity. As parity refers to the number of full term and non-full term pregnancies (miscarriages and premature births), it may suggest that the time of exposure to hormonal changes is determinant for the association.^4^ Also, there is a documented slightly non-significant protective effect of cesarean deliveries,^3^ favoring the hypothesis that the anatomical changes due to labor may maintain the exposition of the transformation zone and enhance the metaplastic process. Finally, studies showed an inconsistent risk of high parity and adenocarcinoma,^2^ ^21^ whose onset could not be explained by the mechanisms presented above.

Screening activities and improvements in development may explain downwards trends in the mortality rates due to period effects, while changes in reproductive factors may explain downwards trends due to cohort effects.^7^ ^8^ Middle- and low-income countries have experienced a substantial decline in fertility rates in the past decades.^19^ A decreasing risk of cervical cancer has been shown in successive generations of women born after 1940 or 1950, in Brazil and in other Latin America countries, in black women in the USA, and in India.^7^

Latin America has the highest socioeconomic disparities in the world, but scarce literature on their relationship with CC control is available. One study from Mexico analyzed factors associated with screening coverage and found that fertility was a better proxy to parity than natality rates, although the variables were not adjusted.^22^ The HDI was negatively associated with mortality rates in the univariate analysis. The HDI is a broad index often used to predict the level of development of regions.^8^ ^10^ A relation between low HDI and high CC incidence and mortality rates was demonstrated in a study that analyzed data from the GLOBOCAN database.^8^ In our study, in the multivariate analysis, the HDI was not significantly associated with mortality rates. As low fertility is recognized as a consequence of socioeconomic development,^19^ probably the role of the HDI on mortality rates is through affecting fertility, or even by enhancing screening programs.

The indicators used to measure access to health care performed better in the South and Southeast regions, and were associated with mortality rates in the univariate analysis. The three federal units with no licensed radiotherapy centers were among the ones with the highest mortality rates. The density of physicians and the density of radiotherapy centers indicate the availability of human and infrastructure resources. Both are influenced by the level of development of the regions. The high proportion of private health insurance observed in federal units with low CC mortality rates can be a marker of a lower efficiency of the public sector when compared with the private sector in terms of cancer screening and care in Brazil.

The availability and efficiency of screening has been strongly associated with declines in CC mortality rates.^6^ ^23^ In our study, we could not demonstrate this association. This could be explained by the weakness of the source used for the indicators, which were based on self-reported data. Brazil does not provide data on the participation of the target population in screening. The self-reported national household survey is the only available data on national coverage. The accuracy of self-reported surveys on screening coverage is limited, since screening histories are usually over-reported by women.^24^ Moreover, in Brazil, it is not clear when women are able to differentiate between pelvic gynecological exam and screening.^25^ In the current situation, it is not possible to monitor and measure the impact of screening interventions.

Low- and middle-income countries suffer from limited availability of data, a barrier to perform studies at the population level. The present study was benefited from the Brazilian national policies to improve the quality of data. By highlighting the differences among the regions, we are suggesting that regional strategies may play an important role when designing a program.

The main limitation of the present study was a weak indicator for screening. Efforts to improve data should be a key point to the program in Brazil. Another limitation was the study design. Ecological studies may suggest associations, but it is not possible to establish a causal effect model only based on its results. However, parity has been consistently associated as a cofactor, so this ecological analysis has to be interpreted in this context. Data regarding the density of radiotherapy centers used for correlation came from the period after the mortality data period, biasing the analysis on temporality. However it was the only data available, and when excluded from the multivariate analysis, the results remained very similar.

## Conclusion

Cervical cancer mortality rates varied in Brazil regarding the place where the women lived. Fertility was the only indicator associated with mortality rates. Accessibility to health care may also have influenced the mortality rates. Information on screening in Brazil should be improved in order to allow analyzing in which extension its actions are contributing for cancer control. Family planning may integrate CC programs in a comprehensive strategy to improve the health of women.
